# Role of oncogenic KRAS in the prognosis, diagnosis and treatment of colorectal cancer

**DOI:** 10.1186/s12943-021-01441-4

**Published:** 2021-11-06

**Authors:** Gongmin Zhu, Lijiao Pei, Hongwei Xia, Qiulin Tang, Feng Bi

**Affiliations:** 1 Department of Abdominal Oncology, Cancer Center and Laboratory of Molecular Targeted Therapy in Oncology, West China Hospital, Sichuan University, No.37 guoxue lane, Chengdu, 610041 Sichuan Province China; 2 The State Key Laboratory of Biotherapy, West China Hospital, Sichuan University, Chengdu, 610041 Sichuan Province China

**Keywords:** Colorectal cancer, KRAS, G12C, Prognosis, Targeted therapy, Combination therapy

## Abstract

Colorectal cancer (CRC) is a heterogeneous disease at the cellular and molecular levels. *Kirsten rat sarcoma* (*KRAS*) is a commonly mutated oncogene in CRC, with mutations in approximately 40% of all CRC cases; its mutations result in constitutive activation of the KRAS protein, which acts as a molecular switch to persistently stimulate downstream signaling pathways, including cell proliferation and survival, thereby leading to tumorigenesis. Patients whose CRC harbors *KRAS* mutations have a dismal prognosis. Currently, *KRAS* mutation testing is a routine clinical practice before treating metastatic cases, and the approaches developed to detect *KRAS* mutations have exhibited favorable sensitivity and accuracy. Due to the presence of *KRAS* mutations, this group of CRC patients requires more precise therapies. However, KRAS was historically thought to be an undruggable target until the development of KRAS^G12C^ allele-specific inhibitors. These promising inhibitors may provide novel strategies to treat *KRAS*-mutant CRC. Here, we provide an overview of the role of KRAS in the prognosis, diagnosis and treatment of CRC.

**Supplementary Information:**

The online version contains supplementary material available at 10.1186/s12943-021-01441-4.

## Introduction

Colorectal cancer (CRC) ranks third in terms of new cases and represents the second leading cause of cancer-related death worldwide in 2018 [1]. Although many advanced management strategies, including improved surgical techniques and , modified adjuvant therapies, and have achieved favorable effects on the treatment of CRC, the mortality of CRC is still high on due to post-operative recurrence and metastasis [2]. CRC is widely considered a heterogeneous disease, with multiple gene alterations and numerous pathways involved in its pathogenesis [3]. The heterogeneity of CRC can be characterized by distinct clinical and pathological features, which lead to diverse prognoses and possibly account, at least in part, for resistance to treatment [4, 5]. Currently, with the rapid development and wide application of next generation sequencing (NGS), molecular profiles of many cancers have been revealed, including CRC, which allows us to use these molecular biomarkers as both predictive and prognostic tools to manage patients with CRC [6].

*Kirsten rat sarcoma* (*KRAS*) is one of the most frequently mutated oncogenes in CRC, with approximately 40% of CRC patients harboring activating missense mutations in *KRAS* and most of them occurring at codons 12, 13 and 61 [7]. Patients with *KRAS*-mutant CRC have a poorer prognosis than those with KRAS-wild-type CRC, especially in the metastatic setting [8, 9]. Moreover, the upstream signal regulation of KRAS is interrupted by aberrant activation of the KRAS pathway, which results in resistance to receptor tyrosine kinase (RTK) inhibitors, such as monoclonal antibodies against epidermal growth factor receptor (EGFR) (cetuximab and panitumumab), in patients with *KRAS*-mutant CRC [10, 11]. Because of the lack of an ideal small molecular binding pocket in the KRAS protein and its high affinity towards abundant guanosine triphosphate (GTP), the development of specific competitive drugs to inhibit *KRAS*-driven oncogenesis has eluded the field. Despite the effort, KRAS is still considered ‘undruggable’, and treatment of *KRAS*-mutant CRC remains a challenge. In recent years, preliminary results from early clinical trials show that direct inhibition of KRAS^G12C^ has become possible, which may provide a novel targeted treatment for a number of patients with advanced CRC [12].

In this review, we briefly describe the role of *KRAS* mutational status in CRC. Then, we summarize the current techniques used to detect *KRAS* mutations. Third, we focus on recent strategies to directly or indirectly inhibit KRAS in CRC, especially breakthrough therapies that target KRAS^G12C^, and detail the clinical use of these inhibitors. Finally, we suggest future directions for the treatment of *KRAS*-mutant CRC.

## KRAS molecular structure and function

The *KRAS* gene encodes a GTP/guanosine diphosphate (GDP)-binding protein that belongs to the guanosine triphosphatase (GTPase) RAS family. The *KRAS* gene alternatively forms two splice variants (*KRAS 4A* and *KRAS 4B*) using different exons 4. Among them, *KRAS 4B* has long been considered the main isoform due to its wide and high expression in human cancers [13, 14]. However, in recent years, *KRAS 4A* was also proven to be ubiquitously expressed in various cancers and able to increase the adaptability of tumor cells under stress [15, 16]. The KRAS protein has a molecular weight of 21 kDa, and is made up of six beta strands and five alpha helices, which form two major domains: the G-domain and the C-terminal [17, 18]. The G domain is highly conserved and contains switch I and switch II loops, which are responsible for GDP-GTP exchange [19]. The C-terminal, a hypervariable region including the CAAX (C = cysteine, A = any aliphatic amino acid, X = any amino acid) motif, is the target for various posttranslational modifications and plays a vital role in newly synthesized and processed KRAS trafficking, as well as final plasma membrane anchoring [13, 19].

**Fig. 1:**
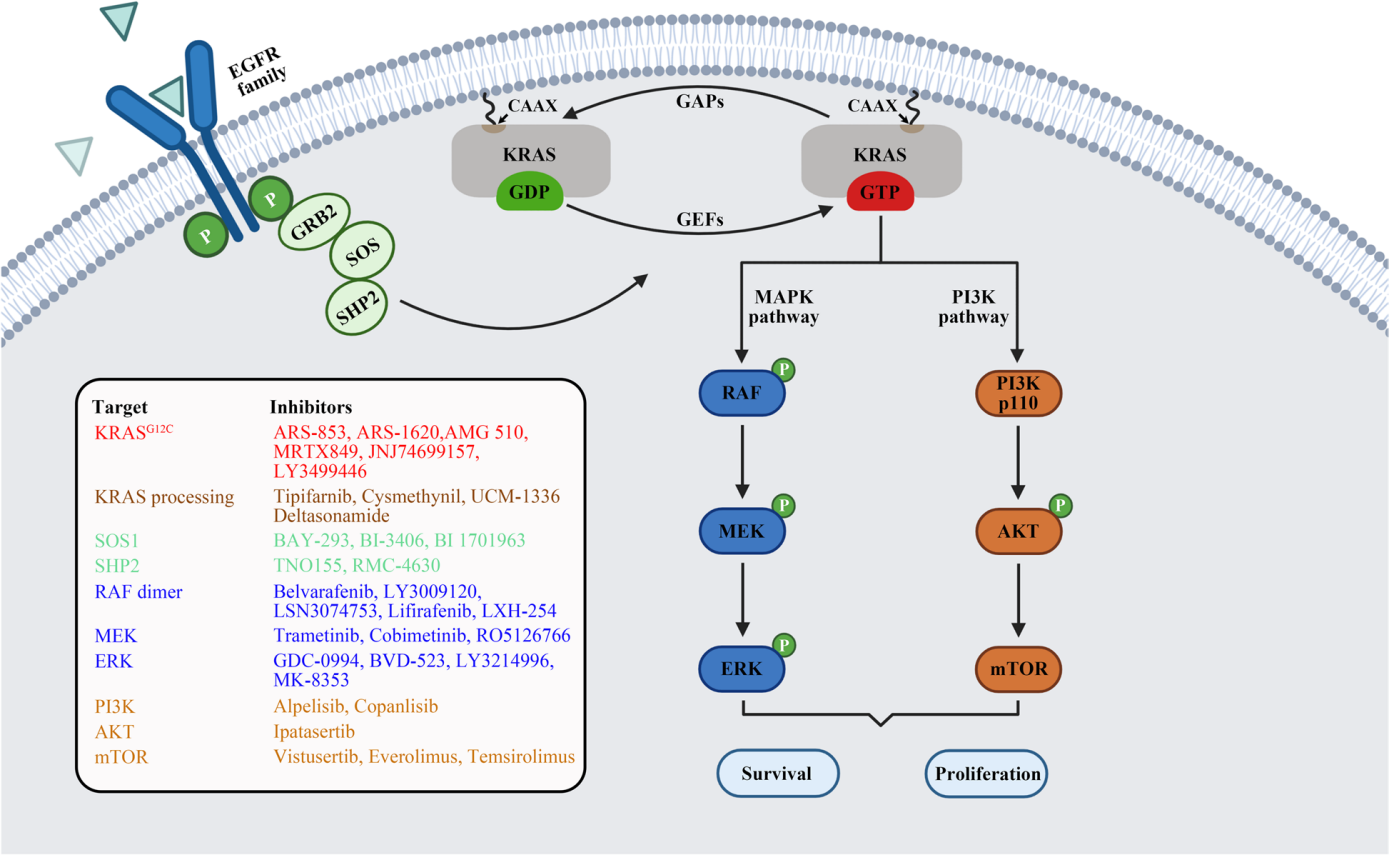
KRAS signaling pathway and relevant inhibitors of each node. After the activation of receptor tyrosine kinase, GRB2 combines with the guanine nucleotide exchange factor SOS and then interacts with KRAS protein that is attached to the cell membrane, thereby promoting KRAS activation. Intrinsic KRAS GTP-GDP cycling is regulated by GEFs and GAPs. Once *KRAS* is mutated, this cycle is disrupted, allowing mutant KRAS protein to accumulate in an active state and thereby persistently activating downstream MAPK and PI3K signaling cascade, resulting in cell proliferation and survival. Various KRAS inhibitors listed in the box were developed to target each node of the KRAS signaling pathway and then evaluated in preclinical or clinical studies. Created with BioRender.com

The KRAS protein acts as a “molecular switch” that cycles between a GDP-bound inactive state and a GTP-bound active state [20]. Although the KRAS protein harbors both intrinsic nucleotide exchange and GTP hydrolysis, its cellular signaling state arises from activation by guanine exchange factors (GEFs), such as son of sevenless (SOS) and Ras guanyl nucleotide-releasing protein, which catalyze GTP loading and deactivation by GTPase activating proteins (GAPs), such as p120GAP and neurofibromin (NF1), which stimulate GTP hydrolysis [21]. GTP binding to KRAS facilitates the binding of effectors to trigger several downstream pathways, including the rapidly accelerated fibrosarcoma (RAF)-mitogen-activated protein kinase kinase (MEK)-extracellular signal-regulated kinase (ERK) and phosphatidylinositol 3-kinase (PI3K)-protein kinase B (AKT)-mechanistic target of rapamycin (mTOR) pathways, which promote cell growth and survival (Fig. 1). In contrast, GDP-bound KRAS loses its activity and prevents its persistent signal transduction activation.

## *KRAS* mutations and their roles in CRC

### *KRAS* mutation subtypes

**Fig. 2:**
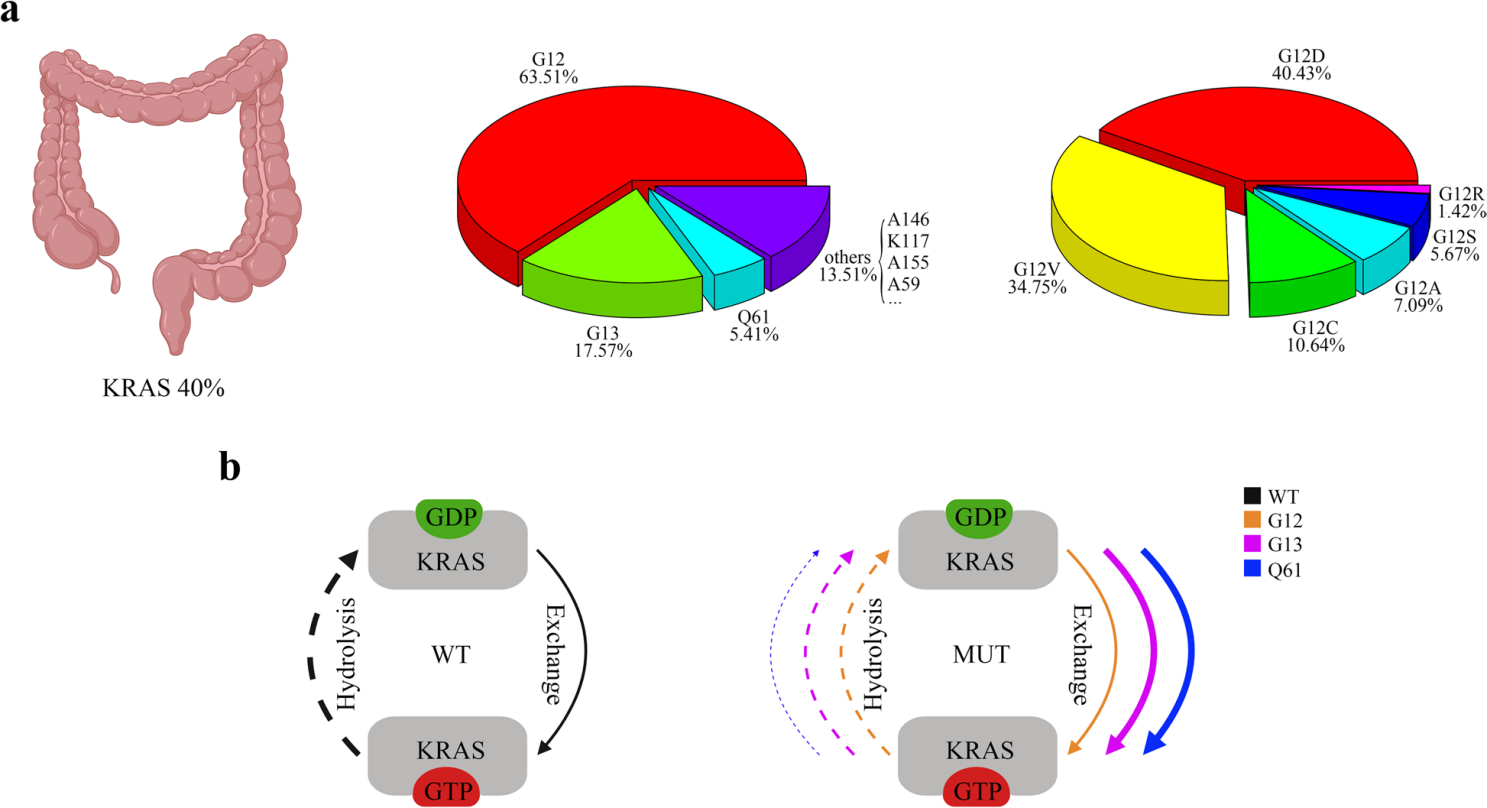
Frequency and distribution of *KRAS* mutations in CRC and the biochemical features of mutant KRAS proteins. **a** Percentage of *KRAS* mutation in CRC and the diversity of *KRAS* alleles. Data acquired from The Cancer Genome Atlas (pan-Cancer) from cBioPortal. **b** Overview of generalized biochemical change of hydrolysis and guanine exchange following mutations in codons 12 (orange), 13 (purple) or, 61 (blue). The dashed line indicates hydrolysis and the solid line indicates guanine exchange, with the thicker line indicating faster rates and vice versa for slower rates. Created with BioRender.com. WT, wild type; MUT, mutant type

*KRAS* is one of the most frequently mutated oncogenes across all malignancies. The prevalence of *KRAS* mutations is approximately 40% in CRC cases (Fig. 2a). Once *KRAS* mutations occur, the hydrolysis of GTP is disrupted and/or nucleotide exchange is enhanced, and then KRAS accumulates in an active state, contributing to continuous activation of downstream signaling pathways, thereby promoting tumor cell proliferation. Therefore, colorectal tumors bearing *KRAS* mutations are associated with advanced disease status, poor tumor differentiation, distant metastasis and inferior survival in patients [9, 22].

In CRC, *KRAS* mutations are most associated with right-sided colon tumors and approximately 85% of *KRAS* mutations occur in one of three major hotspots (codons 12, 13 and 61) [23, 24]. Among them, codon 12 mutation is dominant, accounting for approximately 65% of all *KRAS* alleles. Moreover, G12D (glycine 12 to aspartic acid) and G12V (glycine 12 to valine) are the two most common subtypes in CRC, unlike non-small-cell lung cancer (NSCLC) where G12C (glycine 12 to cysteine) is the most common subtype. The frequency and distribution of *KRAS* mutations for CRC are shown in Fig. 2a. G12 and G13 are located on the P loop, which is required for stabilizing the nucleotide in an active state. Generally, mutations in codon 12 diminish both inherent and GAP-mediated hydrolysis without affecting the rate of nucleotide exchange, except for *KRAS*^*G12C*^, which exhibits GTPase activity similar to that of wild type (Fig. 2b) [25]. In contrast, codon 13 mutations not only decrease hydrolysis but also elevate intrinsic exchange activity (Fig. 2b) [26]. Q61 is located at the N-terminus of switch II and participates in the conformational changes related to this region during the interconversion between structural states. Mutations in codon 61 promote GDP-GTP exchange and simultaneously disrupt GTP hydrolysis (Fig. 2b). Moreover, among all KRAS alleles, Q61 mutants have the lowest hydrolysis rate [25].

### Prognostic and predictive value of *KRAS* mutations

Due to the diversity of *KRAS* alleles in CRC, patients harboring different *KRAS* mutation variants may have a distinct prognosis. Several independent analyses of large cohorts indicated that *KRAS* codon 12 mutations rather than codon 13 mutations were associated with dismal prognosis compared with *KRAS* wild-type cases. When the prognosis was further classified by specific point mutations, G12V and G12C were proven to be correlated with worse overall survival (OS) [27, 28]. Likewise, a pooled analysis including 1239 patients with metastatic CRC from five randomized trials indicated that patients who harbored the *KRAS*^*G12C*^ mutation were associated with worse OS than patients with nonmutated tumors [29]. The reason for the distinction of prognosis is still not well understood. Different biological behaviors of individual mutation variants, such as differential activation of the KRAS downstream effect pathways, have been suggested [30]. However, conflicting views have been proposed, and more frequent *KRAS* variants, such as G12D and G12V, were reported to lack obvious impacts on OS in univariate and multivariate Cox analyses [29]. The clinical data vary among studies, and this may be caused by cohort size, tumor subtyping, tumor staging, genetic background, and even the different methods of collecting mutational data, which makes them difficult to explain and validate.

Chemotherapy based on 5-fluorouracil, leucovorin, and oxaliplatin (FOLFOX) remains the standard first-line treatment for advanced CRC. *KRAS* mutations, especially G12D, are predictive of an inferior response to chemotherapy and a high risk of recurrence [31]. Moreover, *KRAS* mutations are also robust predictors for the efficacy of treatment with EGFR inhibitors in patients with CRC. Monoclonal antibodies targeting ERFR were proven to benefit CRC patients who were refractory to other therapies [32]. However, patients whose tumors harbor *KRAS* mutations in exons 2 (codons 12 and 13), 3 (codons 59 and 61), and 4 (codons 117 and 146) cannot derive benefit from treatment with anti-EGFR therapy, which means that *KRAS* mutations are negative biomarkers for this therapy [33–35]. In fact, not all *KRAS* alleles confer resistance to anti-EGFR therapy. Previous retrospective analyses indicated that CRC patients with the *KRAS*^*G13D*^ mutation benefited from first-line chemotherapy plus cetuximab, although the absolute values of response rate, progression-free survival (PFS) and OS were still below those of patients with *KRAS* wild-type tumors [36, 37].

## Identification of *KRAS* mutations in CRC

Given that oncogenic point mutation of *KRAS* is a common event during CRC and plays a critical role in prognostic evaluation and therapeutic decision-making, *KRAS* mutations should routinely be tested for in the diagnosis of CRC. A variety of laboratory methods are available for the detection of *KRAS* mutations in biological samples, including formalin-fixed paraffin-embedded (FFPE) tissues, fresh tumor tissues, fine-needle aspiration (FNA) materials and cytology and body fluids [38–40]. Detection methods and their sensitivities are summarized in Table 1.

**Table 1:** Summary of the main KRAS detection methods

Techniques	Range of detection	Sensitivity^a^	References
Direct sequencing	All mutations in the interested region	10–30%	[41]
TheraScreen KRAS kit	7 *KRAS* mutations in codons 12 and 13	Approximately 1%	[42, 43]
StripAssay	10 *KRAS* mutations in codons 12 and 13	1%	[44, 45]
SNaPshot	12 *KRAS* mutations in codons 12 and 13	10%	[44, 45]
Cobas	19 *KRAS* mutations in codons 12, 13 and 61	Approximately 1%	[46]
Next generation sequencing	All clinical relevant *KRAS* mutations	1–6%	[47, 48]
Droplet digital PCR	7 *KRAS* mutations in codons 12 and 13	0.01–0.05%	[48, 49]
BEAMing	16 *KRAS* mutations in codons 12, 13, 59, 61, 117 and 146	0.01%	[48, 50]

Abbreviations: *BEAM* beads, emulsion, amplification, magnetics

^a^Sensitivity is the mutant to wild-type ratio showed as a percentage

### Routine laboratory methods

Currently, direct sequencing, namely, polymerase chain reaction (PCR) followed by dideoxy sequencing, is still the gold standard method for detecting mutations. This method is able to detect all mutations in the region of interest, but it requires a high allele frequency of mutation (10–30%) to meet the level of detection, suggesting that it may not be appropriate for clinical application due to its sensitivity [41, 42].

TheraScreen KRAS kit (Qiagen), a test based on amplification refractory mutation system (ARMS) technology, is the first clinically validated and FDA-approved kit widely used to evaluate tumor-specific mutations in patients with CRC [43]. This kit detects seven mutations in codons 12 and 13 in the *KRAS* oncogene, and has higher sensitivity and specificity than direct sequencing. In addition, this kit has been applied to phase III clinical trials for metastatic CRC [11, 44]. StripAssay (Vienna Labs) based on mutant-enriched PCR followed by reverse hybridization also has a much lower detection threshold than direct sequencing, and it can detect 10 of the most common mutations (eight in codon 12 and two in codon 13). However, its flexibility is poor, and the cost is much higher than that of direct sequencing. Another technique, SNaPshot, is not as sensitive as StripAssay, but it can detect 12 mutations in codons 12 and 13, and it is more flexible and cheaper than StripAssay [45]. The TaqMelt PCR assay Cobas (Roche) can detect 19 *KRAS* mutations in codons 12, 13 and 61 and is more sensitive than the TheraScreen assay. Moreover, this assay is highly reproducible (> 98%) and has a rapid turnaround time (1 day) [46].

However, the limitation of these kits is the inability to detect uncommon mutated alleles, and patients with these mutations hardly benefit from anti-EGFR therapies. Next generation sequencing, due to its great sensitivity and entire exon sequencing, can identify all clinically relevant *KRAS* mutations [47]. NGS is based on the original concept of pyrosequencing but uses fluorescence markers or pH measurements to determine the sequence of DNA nucleotides. This technology is now well established and is routinely used to analyze mutations in solid and liquid (hematologic) samples in many laboratories. Due to the high cost of NGS per sample, NGS panels for CRC usually analyze mutational hotspots in various oncogenes, which provides broader views for the occurrence and progression of tumors and is more likely to find druggable targets than only detect *KRAS* [51].

### *KRAS* mutation assay in liquid biopsy samples

Currently, the detection of *KRAS* mutations is most commonly carried out in tumor tissue, especially FFPE tumor tissues. Although these analyses based on tumor tissues acquire satisfactory results in terms of sensitivity and specificity, they rely heavily on the quality and quantity of the tumor samples and have a slow turnaround time [52], which may not meet the urgent requirement of patients in first-line treatment for metastatic CRC [53]. Liquid biopsy, an emerging analytical technique, has the advantages of minimal invasiveness, rapid detection, and the ability to present comprehensive molecular characteristics of the disease, enabling early diagnosis, evaluation of the response to molecular targeted therapies and early exploration of the potential resistance mechanisms of cancer cells [54]. Likewise, the clinical application of liquid biopsy has also been developed in each clinical stage of CRC, of which circulating tumor DNA (ctDNA) is the most clinically advanced. ctDNA is cell-free DNA (cfDNA) released into circulation by tumor cells and reflects mutations specific to tumor cells [55]. It can be isolated from plasma but only accounts for a small part of the total cfDNA isolated from plasma. For this reason, highly sensitive techniques are required for the ctDNA test. This requirement is currently met by the development of PCR-based methods, such as allele-specific quantitative PCR-based Intplex technology and emulsion PCR techniques (ddPCR and BEAMing), and the advent of NGS [48–50, 52]. Therefore, it is no surprise that *KRAS* mutation detection in blood from patients with CRC is gaining momentum.

Nevertheless, despite the use of highly sensitive technology, circulating *KRAS* mutation detection does not perfectly reflect the mutation burden of the primary tumor from which it originated. One might speculate that tumor cells at an early stage cannot release adequate ctDNA or that the concentration of ctDNA is low and its quality degraded. In addition, several studies have reported the discordance between ctDNA and tissues in examining *KRAS* mutations in CRC patients [56, 57]. On the one hand, ctDNA tests are able to detect shed DNA from various tumor sites in theory, whereas tissue biopsy only finds alterations at the specific site of sampling. On the other hand, the difference in sensitivity of nucleic acid processing and analytical technologies may also lead to discordance.

Overall, liquid biopsy is a promising field in CRC, whereas the clinical use of this approach still requires large prospective studies with adequate cohorts of patients and standardized methods of analysis. But anyway, one point is clear that liquid biopsy *KRAS* testing can provide complementary information to the tissue tests [54].

## KRAS targeting therapy in CRC

### Historical perspectives on KRAS targeting therapy

For a long time, KRAS has been considered an “undruggable” target due to the specific characteristics of KRAS its molecular structure. The KRAS protein is a small protein with a relatively smooth surface. In addition to the GTP/GDP binding pocket, the KRAS protein does not provide enough pockets for small molecular inhibitor binding [58]. Moreover, the extremely high affinity of KRAS for GTP and the high cellular concentrations of GTP make it almost impossible to develop a competitive small molecule inhibitor [58]. Additionally, indiscriminate inhibition of both wild-type and mutant KRAS may lead to potential toxicity [59]. Therefore, inhibiting KRAS directly is a great challenge for *KRAS*-mutant tumor treatment.

Similarly, approaches that indirectly target KRAS, including the inhibition of nucleotide exchange, processing, membrane localization and molecules in the signaling pathway, have not been very effective clinically. Multiple positive and negative feedback loops implicated in the KRAS signaling network enable easy rebound of the therapeutic [60, 61]. In addition, the mutant KRAS protein can activate other cancer-related cellular processes, such as cell Warburg metabolism to maintain tumor growth, which results in the low or absent inhibitory effect of the indirect targeting of KRAS [62].

Although KRAS-targeting therapy is still an enormous challenge, various attempts have been made to discover small molecules to overcome this problem. Here, we highlight the latest efforts in targeting mutant *KRAS* and summarize the relevant clinical trials conducted in CRC (Tables 2 and 3) and the characteristics of some of these drugs (Additional file 1: Table S1).

**Table 2:** Single-agent therapies in clinical trials

Inhibitor	Biomarker	Cancer type	CRC patients enrollment	Phase	NCT number	Status
**KRAS**^**G12C**^ **inhibitors**						
AMG 510	*KRAS*^*G12C*^ mutation	Advanced solid tumors	Enrolled	I/II	NCT03600883	Recruiting
MRTX849	*KRAS*^*G12C*^ mutation	Advanced solid tumors	Enrolled	I/II	NCT03785249	Recruiting
JNJ-74699157	*KRAS*^*G12C*^ mutation	Advanced solid tumors	Enrolled	I	NCT04006301	Completed
LY3499446	*KRAS*^*G12C*^ mutation	Advanced solid tumors	Enrolled	I/II	NCT04165031	Terminated
**SOS inhibitors**						
BI 1701963	*KRAS* mutations	Advanced solid tumors	Not mentioned	I	NCT04111458	Recruiting
**SHP2 inhibitors**						
RMC-4630	Mutations that hyperactive the RAS-MAPK pathway	Relapsed or refractory solid tumors	Not mentioned	I	NCT03634982	Recruiting
**RAF inhibitors**						
LY3009120	*BRAF*, *NRAS* or *KRAS* mutations	Advanced or metastatic solid tumors	Enrolled	I	NCT02014116	Terminated
BGB-283	*BRAF*, *NRAS* or *KRAS* mutations	Advanced solid tumors	Enrolled	I	NCT02610361	Completed
**ERK inhibitors**						
GDC-0994	*BRAF* or *KRAS* mutations	Advanced or metastatic solid tumors	Enrolled	I	NCT01875705	Completed
BVD-523	*NRAS, HRAS, KRAS, BRAF, MEK* or *ERK* mutations	Advanced MAPK pathway-altered malignancies	Enrolled	I	NCT04566393	Available
**Adoptive cell therapies**						
Anti-KRAS G12D mTCR	HLA-A*11:01 positive *KRAS*^*G12D*^ mutation	Advanced solid tumors	Enrolled	I/II	NCT03745326	Suspended
Anti-KRAS G12V mTCR	HLA-A*11:01 positive *KRAS*^*G12D*^ mutation	Advanced solid tumors	Enrolled	I/II	NCT03190941	Suspended
**Cancer vaccines**						
TG02	*KRAS* exon 2, codon 12 or 13 mutation	Rectal cancer	Enrolled	I	NCT02933944	Terminated
mRNA-5671	*KRAS*^*G12D*^, *KRAS*^*G12V*^, *KRAS*^*G12C*^, *KRAS*^*G13D*^ mutation	NSCLC, non-MSI-H CRC, PDAC	Enrolled	I	NCT03948763	Recruiting

Abbreviations: *CRC* colorectal cancer, N*SCLC* non-small cell lung cancer, *PDAC* pancreatic ductal adenocarcinoma, *MSI-H* microsatellite instability-high, *TCR* T-cell receptor, *HLA* human leukocyte antigen. Data acquired from ClinicalTrials.gov

**Table 3:** Combination therapies in clinical trials

Inhibitor	Biomarker	Cancer type	CRC patients enrollment	Phase	NCT number	Status
**KRAS**^**G12C**^ **combinations**						
AMG 510 and panitumumab and FOLFIRI regimen	*KRAS*^*G12C*^ mutation	Advanced solid tumors	Enrolled	Ib/II	NCT04185883	Recruiting
AMG 510 and trametinib and panitumumab	*KRAS*^*G12C*^ mutation	Advanced solid tumors	Enrolled	Ib/II	NCT04185883	Recruiting
AMG 510 and MVASI® (bevacizumab-awwb) and FOLFIRI or FOLFOX regimen	*KRAS*^*G12C*^ mutation	Advanced solid tumors	Enrolled	Ib/II	NCT04185883	Recruiting
MRTX849 and TN0155	*KRAS*^*G12C*^ mutation	Advanced solid tumors	Enrolled	II	NCT04330664	Recruiting
MRTX849 and cetuximab	*KRAS*^*G12C*^ mutation	Advanced or metastatic CRC	Enrolled	III	NCT04793958	Recruiting
MRTX849 and mFOLFOX6 regimen	*KRAS*^*G12C*^ mutation	Advanced or metastatic CRC	Enrolled	III	NCT04793958	Recruiting
MRTX849 and FOLFIRI regimen	*KRAS*^*G12C*^ mutation	Advanced or metastatic CRC	Enrolled	III	NCT04793958	Recruiting
**SOS inhibitors combinations**						
BI 1701963 and trametinib	*KRAS* mutations	Advanced solid tumors	Not mentioned	I	NCT04111458	Recruiting
**SHP2 inhibitors combinations**						
RMC-4630 and LY3214996	*KRAS* mutations	Advanced or metastatic solid tumors	Enrolled	I	NCT04916236	Not yet recruiting
TNO155 and JDQ443	*KRAS*^*G12C*^ mutation	Advanced solid tumors	Enrolled	Ib/II	NCT04699188	Recruiting
**RAF-MEK-ERK inhibitors combinations**						
HM95573 and cobimetinib	*RAS* or *RAF* mutations	Advanced or metastatic solid tumors	Enrolled	I	NCT03284502	Recruiting
GDC-0994 and cobimetinib	None	Advanced or metastatic solid tumors	Enrolled	Ib	NCT02457793	Completed
MK-8353 and pembrolizumab	None	Advanced solid tumors	Enrolled	Ib	NCT02972034	Active, not recruiting
**Cancer vaccines combinations**						
TG02 and pembrolizumab	*KRAS* exon 2, codon 12 or 13 mutation	Rectal cancer	Enrolled	I	NCT02933944	Terminated
mRNA-5671 and pembrolizumab	*KRAS*^*G12D*^, *KRAS*^*G12V*^, *KRAS*^*G12C*^, *KRAS*^*G13D*^ mutation	NSCLC, non-MSI-H CRC, PDAC	Enrolled	I	NCT03948763	Recruiting

Abbreviations: *CRC* colorectal cancer, *NSCLC* non-small cell lung cancer, *PDAC* pancreatic ductal adenocarcinoma, *MSI-H* microsatellite instability-high, *FOLFOX* 5-fluorouracil, leucovorin, and oxaliplatin, FOLFIRI: 5-fluorouracil, leucovorin, and irinotecan. Data acquired from ClinicalTrials.gov

### Strategies to target KRAS directly

#### Covalent inhibitors targeting KRAS^G12C^

Recently, the discovery of inhibitors that selectively target KRAS^G12C^ while preserving wild-type or other mutant KRAS to circumvent the toxicity caused by inhibition of all KRAS isoforms was a breakthrough in this field of research [63]. KRAS^G12C^ arises from a glycine-to-cysteine substitution at codon 12, and the thiol group in the cysteine residue is an attractive target for covalent inhibitors. In contrast, wild-type KRAS is not inhibited by this covalent approach due to the lack of cysteine in the active site. The first series of compounds targeting KRAS^G12C^ was developed by Shokat and colleagues. These compounds covalently bind to the mutant cysteine residue and occupy a new allosteric pocket behind switch-II, namely, the switch-II pocket, when KRAS^G12C^ is in its GDP-bound state [63]. Then, these compounds change the nucleotide preference of KRAS from GTP to GDP, which contributes to the accumulation of KRAS in the inactive state. Moreover, these compounds attenuate associations with regulatory proteins and effectors such as the exchange factors SOS and RAF, and thereby prevent signaling by KRAS. However, the most potent compound developed by Shokat and colleagues (Compound 12) was reported to have low pharmacological properties [64].

**Fig. 3:**
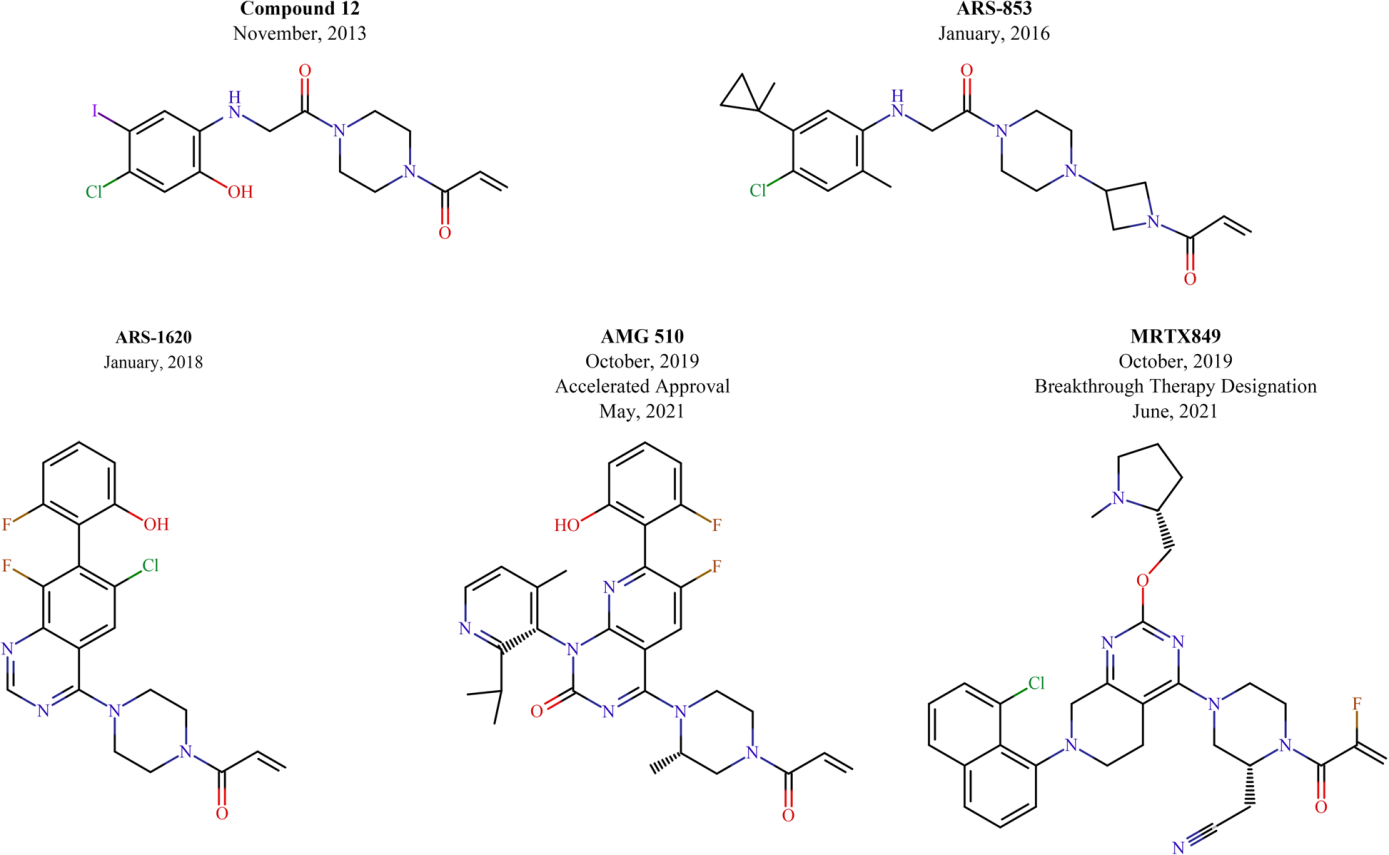
Chemical structures of KRAS^G12C^ covalent inhibitors with their initial publication date. AMG 510 has received accelerated approval from the U.S. FDA for the treatment of patients with NSCLC in May 2021. MRTX849 has been granted Breakthrough Therapy Designation by FDA in June 2021

Subsequently, numerous studies have continued to improve the efficacy of KRAS^G12C^ inhibition, and a series of novel KRAS^G12C^ inhibitors have been developed, including ARS-853 [64], ARS-1620 [65], AMG 510 [66] and MRTX859 [67, 68] (Fig. 3). Among them, AMG 510 was the first compound to enter clinical trials and showed remarkable single-agent efficacy in a phase I trial, especially in NSCLC (NCT03600883; Table 2). Recently, LUMAKRAS™ (sotorasib), also known as AMG 510, received accelerated approval from the U.S. Food and Drug Administration (FDA) for the treatment of adult patients with *KRAS*^*G12C*^-mutant locally and metastatic NSCLC who have received at least one prior systemic therapy [69]. The emergence and development of AMG 510 were attributed to the discovery of a surface groove created by an alternative orientation of histidine residue (His95), which could be occupied by aromatic rings and which significantly enhanced its interactions with the KRAS^G12C^ protein [70]. Therefore, AMG 510, as a His95-binding molecule, is approximately 10 times more effective than ARS-1620, although they are structurally related and overlap [66]. In preclinical models, AMG 510 was able to induce *KRAS*^*G12C*^-mutant colorectal cancer patient-derived xenograft (CRC PDX) shrinkage. Additionally, in mice bearing CT-26 *KRAS*^*G12C*^ tumor, AMG 510 also led to tumor regression [66]. In the first human study (NCT03600883), a total of 129 patients with *KRAS*^*G12C*^-mutant cancers (59 NSCLC, 42 CRC and 28 other tumors) were enrolled in the study. Among them, NSCLC showed the highest response rate to AMG 510: across all dose levels, 32.2% (19 patients) had an objective response, 88.1% (52 patients) had disease control, and the median PFS for all NSCLC patients was 6.3 months. However, the activity in CRC was less promising: 7.1% (3 patients) had an objective response, 73.8% (31 patients) had disease control, and the median PFS for all CRC patients was 4.0 months [12]. Notably, no dose-limiting toxicities have been observed with AMG 510, even with extended treatment. In consideration of the poor efficacy of AMG 510 as monotherapy in patients with CRC, combination therapies are being explored. A previous study found that the immune system plays a critical role in the long-term cure induced by AMG 510 in the CT-26 *KRAS*^*G12C*^ model. Subsequently, AMG 510 treatment was found to induce an inflamed tumor microenvironment and led to increased infiltration and activation of T cells, which provided a theoretical basis for its combined immunotherapy. Indeed, combination with anti-programmed cell death 1 (PD-1) therapy enhanced the durability of regression of CT-26 *KRAS*^*G12C*^ tumors and significantly improved survival [66]. Amodio and colleagues found that *KRAS*^*G12C*^-mutant CRC cell lines displayed higher basal RTK activation, especially EGFR, than NSCLC cell lines; they then revealed that the reactivation of EGFR reversed the efficacy of AMG 510 in CRC. Therefore, concomitant EGFR inhibition is required to acquire a better response after KRAS^G12C^ inhibition [71]. Currently, AMG 510 in combination with other inhibitors and chemotherapies is under clinical investigation (NCT04185883; Table 3).

MRTX849 is another potently covalent KRAS^G12C^ inhibitor. On June 24, 2021, the U.S. FDA granted Breakthrough Therapy Designation to adagrasib (MRTX849) for the potential treatment of patients with *KRAS*^*G12C*^-mutant NSCLC following prior systemic therapy [72]. In preclinical models, MRTX849 showed remarkable anti-tumor efficacy exclusively in *KRAS*^*G12C*^-mutant cell lines and resulted in tumor regression in xenograft models [67]. Further study indicated that the anti-tumor activity of MRTX849 was significantly improved when combined with upstream (EGFR and SHP2) inhibitors and downstream (mTOR and cyclin-dependent kinase 4/6) (CDK4/6) inhibitors [67]. A phase I/II clinical trial of MRTX849 is also ongoing (NCT03785249; Table 2). The updated clinical data of adagrasib were presented at the 2020 EORTC-NCI-AACR International Virtual Conference on Molecular Targets and Therapeutics. As of August 30, 2020, among the evaluable efficacy data in heavily pretreated patients with advanced NSCLC (*n* = 51) treated with MRTX849 as a monotherapy at a 600 mg BID dose, 45.1% (23 patients) had a confirmed objective response, and 96.1% (49 patients) had disease control. However, the clinical activity in CRC (*n* = 18) was inferior to that in NSCLC: 16.7% (3 patients) had an objective response, and 94.4% (17 patients) had disease control [73]. Therefore, to improve the efficacy of MRTX849 in CRC, trials of its combination of cetuximab (an anti-EGFR antibody) and TNO155 (an SHP2 inhibitor) are underway, but the results have not been published (NCT04793958, NCT04330664; Table 3). Similarly, MRTX849 was well-tolerated across monotherapy and combination trials.

A third KRAS^G12C^ covalent inhibitor, JNJ-74699157 (ARS-3248), is also being evaluated in a phase I clinical trial (NCT04006301; Table 2). Although the results of the clinical trial have not been published, its predecessor compounds (ARS-853 and ARS-1620) are able to induce tumor regression in PDX models and block downstream signaling to mitogen-activated protein kinase (MAPK) in *KRAS*^*G12C*^-mutant tumor cell lines [65, 74]. Another KRAS^G12C^ covalent inhibitor developed by Eli Lilly, LY3499446, has been under clinical investigation (NCT04165031; Table 2) and assessed as a monotherapy and in combination with other inhibitors, including cetuximab, abemaciclib and erlotinib, or with chemotherapy (docetaxel) in advanced solid tumors, including NSCLC and CRC. However, this clinical trial was terminated due to unexpected toxicity.

#### Targeting the RAS-binding pocket

Although covalent inhibitors of KRAS^G12C^ targeting the mutation-specific state are effective, identifying effective treatments for each mutant KRAS protein is cumbersome. Fang, Wang, Fesik, and colleagues identified a series of compounds that could interact with the hydrophobic pocket on the RAS-GDP complex and then block the RAS-SOS interaction, thus inhibiting SOS-mediated nucleotide exchange [75, 76]. In addition, researchers at Kobe University adopted different approaches and made efforts to explore small molecules that could interfere with the interaction between GTP-bound RAS and RAF. Kobe0065, the most promising compound they developed, was proven to bind to RAS-GTP (HRAS and KRAS) and competitively block RAF binding, which inhibited the growth of cancer cells carrying activated *RAS* oncogenes and tumor xenografts [77].

These compounds function as pan-RAS inhibitors. Therefore, the low specificity of these compounds may pose toxicity problems. Additionally, their low potency in preclinical models may limit their value in clinical applications. Despite the deficiencies of these compounds, they question the theory that KRAS is an “undruggable” target.

### Strategies to target KRAS indirectly

Nucleotide exchange, KRAS processing, and membrane localization are all involved in KRAS activation. Meanwhile, the activation of the KRAS downstream signaling pathways also drives KRAS-mediated oncogenesis. Therefore, interference with any of the above steps can indirectly affect KRAS function.

#### Inhibitors of the nucleotide exchange cycle

##### SOS1 inhibitors

In addition to the previously described method of targeting the hydrophobic pocket of the RAS-GDP complex to inhibit SOS binding, direct inhibition of SOS1 is an effective approach to inhibit GEF activity. Several studies described that a lipophilic pocket of SOS1 adjacent to the RAS-binding site could be occupied by small molecules, which could activate or block the SOS-RAS interaction [78, 79]. BAY-293, one of these small molecules, is a selective SOS1 inhibitor that can inhibit the RAS-RAF-MEK-ERK pathway. Additionally, when combined with a KRAS^G12C^ covalent inhibitor, BAY-293 demonstrated synergistic antiproliferative activity in a *KRAS*^*G12C*^-mutant cell line, due to its ability to increase the pool of GDP-bound KRAS^G12C^ [79]. However, BAY-293 did not show the expected differential effect on *KRAS*-mutant cancer cell lines versus wild-type *KRAS* cells. Recently, BI-3406, a more potent and selective SOS1 inhibitor, was developed. BI-3406 is the first orally bioavailable inhibitor of the SOS1-KRAS interaction, and it only inhibits SOS1 and not SOS2 [80]. In *KRAS*-mutant cancer, whether G12 or G13, BI-3406 decreased the KRAS-GTP level and restricted the proliferation of the majority of tumor cells. Currently, BI 1701963, a BI-3406 analogue, is being evaluated in a phase I clinical trial alone or in combination with trametinib in patients with advanced *KRAS*-mutant tumors (NCT04111458; Tables 2 and 3).

##### SHP2 inhibitors

SHP2 is a protein tyrosine phosphatase encoded by the gene *PTPN11*, and its tyrosine phosphorylation contributes to interaction with growth factor receptor-bound protein 2 (GRB2). Thus, it has been speculated that SHP2 acts as a scaffold protein to recruit the GRB2-SOS complex, thereby promoting RAS nucleotide exchange [81, 82]. Therefore, the effect of inhibiting SHP2 is similar to that of SOS1 inhibitors, which block the loading of GTP on wild-type RAS. A previous study demonstrated that SHP2 inhibitors limited the proliferation of *KRAS*-mutant CRC in vitro and in vivo [83]. More importantly, several studies have indicated that reversing adaptive resistance to MEK inhibitors is a more effective application of SHP2 inhibitors in *KRAS*-dependent cancers [84–86]. Nevertheless, the tolerability of this combination is a concern. A recent study suggested that the CDK4/6 inhibitor ribociclib could be used as an alternative to the MEK inhibitor trametinib to improve the efficacy of the SHP2 inhibitor TNO155 in *KRAS*-mutant CRC and showed better tolerability. Moreover, this study also indicated that TNO155 could improve the efficacy of KRAS^G12C^ covalent inhibitors against *KRAS*^*G12C*^-mutant CRC [87]. In addition to tumor intrinsic effects, SHP2 inhibitors also have immunomodulatory effects. In CT-26 and MC38 CRC models, SHP2 inhibitors induce a reduction in protumorigenic M2 macrophages and can improve the efficacy of PD-1 blockade [87, 88]. Currently, there are several SHP2 inhibitors in the early phase of clinical trials. RMC-4630 is in a phase I clinical trial as a single agent (NCT03634982; Table 2) and in a new clinical trial in combination with an ERK inhibitor, LY3214996, for the treatment of patients with metastatic *KRAS*-mutant cancers (NSCLC, CRC and pancreatic cancer) (NCT04916236; Table 3). A second inhibitor, TNO155, is in a phase Ib/II clinical trial in combination with the KRAS^G12C^ inhibitor JDQ443 in *KRAS*^*G12C*^-mutant cancers, including CRC (NCT04699188; Table 3).

#### Disruption of KRAS processing and membrane localization

Localization to the cell membrane is required for KRAS GTP binding and activation, so interference with KRAS membrane association may be a potential approach for targeting KRAS. Post-translational modification of KRAS is the first step of membrane localization, and there are three key enzymes involved in this process: farnesyltransferase (FTase) or geranylgeranyltransferase (GGTase), RAS-converting enzyme (RCE1) and isoprenylcysteine carboxyl methyltransferase (ICMT). Consequently, inhibitors of these enzymes have been developed to attenuate KRAS activity. However, in a preclinical study, *KRAS*-mutant cell lines were found to be less sensitive to the FTase inhibitor tipifarnib than *HRAS*-mutant cell lines [89]. Similarly, tipifarnib showed disappointing effects in phase II and III clinical trials on patients with advanced CRC [90, 91]. These results may be due to the functional redundancy between FTase and GGTase. Targeting the downstream RAS processing enzymes, namely, RCE1 and ICMT, is an effective approach to prevent the compensation of FTase inhibitors by GGTase in *KRAS*-mutant tumors. Inhibitors of ICMT, such as cysmethynil and UCM-1336, have been developed and have shown a great capability to reduce KRAS activity and impair the growth of *KRAS*-mutant cell lines [92, 93]. However, the clinical data of ICMT inhibitors in patients with KRAS-mutant CRC are not yet available.

Post-translationally modified KRAS requires the regulation of prenyl-binding protein phosphodiesterase-δ (PDEδ) to transport and localize to the membrane [94]. Deltarasin, a high-affinity PDEδ-RAS interaction inhibitor, blocks KRAS binding by occupying the farnesyl-binding pocket of PDEδ and thereby leads to mislocalization of KRAS [95]. In CRC cell lines, deltarasin specifically inhibits the proliferation of cell lines with oncogenic *KRAS* mutations, and the latest generation of the PDEδ inhibitor, deltasonamide 2, shows a more superior suppression effect [96]. However, these inhibitors are quickly released from PDEδs induced by Arl2, which hampers their anti-tumor efficacy [97]. Recently, to overcome this limitation, the first-in-class PDEδ degraders were designed by the proteolysis-targeting chimeric (PROTAC) method from conventional PDEδ inhibitors to induce PDEδ degradation. Subsequently, Compound 17f, the most promising PDEδ degrader, shows enhanced anti-tumor activity in the *KRAS*-mutant CRC cell line and its xenograft model [98].

Notably, the enzymes mentioned above also process other membrane-associated proteins, which may result in off-target effects.

#### Targeting downstream signaling pathways

##### RAF-MEK-ERK inhibitors

KRAS activation accelerates dimerization and phosphorylation of its downstream RAF proteins, which induces RAF kinase activity and then contributes to phosphorylation of RAF substrates MEK1/2. The phosphorylation cascade continues followed by MEK phosphorylation of the terminal kinase, ERK1/2. Ultimately, activation of ERK1/2 results in activating transcription factors that regulate genes promoting cell growth and preventing cells from undergoing apoptosis [99]. However, the RAF-MEK-ERK cascade is not a linear and unidirectional signaling pathway, as it has multiple inputs and outputs, feed-forward and feedback mechanisms, and multiple scaffold proteins that dynamically regulate signaling and ERK activity [100]. Therefore, only almost complete inhibition of the RAF-MEK-ERK pathway can effectively treat *KRAS*-mutant tumors.

RAF dimerization mediated by active KRAS, leads to the activation of RAF. LY3009120, an RAF dimer inhibitor, effectively inhibits the activities of the BRAF/CRAF heterodimer and BRAF or CRAF homodimer by occupying both promoters in the dimer, although it can also promote dimerization [101]. In a preclinical setting, LY3009120 displayed an anti-proliferative effect in *KRAS*-mutant CRC cell lines and inhibited tumor growth in *KRAS*-mutant xenograft models [102]. Furthermore, its analog, LSN3074753, showed synergistic antitumor activity in *KRAS*-mutant CRC PDX models when combined with cetuximab [103]. A phase I clinical trial of LY3009120 has been conducted in patients with advanced solid tumors, but the clinical efficacy was extremely limited in CRC patients with the *KRAS* mutation (NCT02014116; Table 2) [104]. Similarly, another RAF dimer inhibitor, lifirafenib (BGB-283), also showed no responses to patients with *KRAS*-mutant CRC in a phase I clinical trial (NCT02610361; Table 2) [105].

MEK inhibitors as single agents have shown disappointing results in *KRAS*-mutant tumors, especially in *KRAS*-mutant CRC [106, 107]. This is due to feedback-mediated RAF activation leading to an increase in phosphorylated MEK when *KRAS*-mutant tumors are treated with MEK inhibitors, which contributes to weak efficacy in these tumors [108, 109]. Therefore, no MEK inhibitor is clinically approved for the treatment of *KRAS*-mutant tumors. Although both MEK and RAF inhibitor monotherapy are less promising in *KRAS*-mutant CRC, the combination of these two inhibitors demonstrated synergy in preclinical models of *KRAS*-mutant CRC cell lines [110, 111]. Currently, a phase I clinical trial investigating the combination of belvarafenib (HM95573, RAF inhibitor) and cobimetinib (MEK inhibitor) is recruiting patients with locally advanced solid tumors, including CRC (NCT03284502, Table 3). In addition, concomitant blockade of MEK and KRAS exhibits synergistic anti-tumor effect in *KRAS*-mutant CRC, which can provide a theoretical basis for the combination of MEK inhibitors and KRAS^G12C^ covalent inhibitors in treating *KRAS*^*G12C*^*-*mutant CRC [112, 113].

As ERK is the culminating kinase of this pathway, it has been speculated that inhibition of ERK may provide an effective therapeutic option for *KRAS*-mutant tumors. However, ERK inhibitors, such as RAF and MEK inhibitors, have shown poor results in clinical trials treating patients with *KRAS*-mutant tumors, especially those with *KRAS*-mutant CRC. GDC-0994, an ERK inhibitor, was evaluated as a single agent in a phase I clinical trial. In this trial, 5 patients with *KRAS*-mutant CRC were enrolled and treated with GDC-0994, of which only one achieved stable disease and the remainder had progressive disease (NCT01875705; Table 2) [114]. However, when in combination with a MEK inhibitor, GDC-0994 exhibited enhanced anti-tumor efficacy in *KRAS*-mutant CRC cell lines [115]. Moreover, GDC-0994 is being evaluated in a phase Ib clinical trial in combination with cobimetinib for the treatment of advanced solid tumors (NCT02457793; Table 3). Other ERK inhibitors, including BVD-523, LY3214996 and MK-8353, are in the early stage of clinical trials either alone or in combination (NCT04566393; Table 2; NCT04916236, NCT02972034; Table 3).

In general, the efficacy of RAF, MEK or ERK inhibitors alone in *KRAS*-mutant CRC is not satisfactory. Therefore, the focus should be placed more on exploring the use of these inhibitors in combination with other inhibitors of the MAPK pathway or with other inhibitors that we mentioned in this Review, which may bring surprise for the treatment of *KRAS*-mutant CRC.

##### PI3K-AKT-mTOR inhibitors

In addition to the RAF-MEK-ERK pathway, PI3K is the second effector pathway that is also activated by KRAS. GTP-bound KRAS stimulates PI3Ks to convert phosphatidylinositol-4,5-bisphosphate to phosphatidylinositol-3,4,5-trisphosphate, which contributes to the recruitment of AKT to the cell membrane where it becomes phosphorylated and then activates mTOR. Therefore, inhibition of this pathway at each node may be an optional approach for the treatment of *KRAS*-mutant tumors. However, most monotherapies targeting PI3K, AKT, and/or mTOR are ineffective in *KRAS*-mutant cancer [116].

PI3K pathway inhibitors are more suitable as an option in combination therapy for *KRAS*-mutant cancers, because there are overlapping feedback mechanisms between the MAPK and PI3K pathways, meaning that inhibition of one pathway can result in the compensatory activation of the other [111, 117, 118]. Therefore, the combination of MAPK and PI3K inhibitors may be a compelling regimen for the treatment of *KRAS*-mutant cancers. In *KRAS*-mutant CRC cell lines, PI3K pathway inhibitors overcome resistance to MEK inhibitors and significantly inhibit cell proliferation. Moreover, this robust synergistic activity has also been observed in mouse models [117, 119]. However, in clinical trials, these combined treatment strategies appear to be ineffective in patients with *KRAS*-mutant CRC, which may be the result of dose limitation due to toxicity [120–122].

Furthermore, upregulation of the PI3K pathway can also occur through activating mutations in *PIK3CA*, which encodes catalytic p110α kinase activity. This mutation can coexist with *KRAS* mutations, which suggests that inhibitors targeting KRAS are sufficient to inhibit the MAPK pathway rather than the PI3K pathway [123, 124]. Therefore, the combination of PI3K inhibitors and other inhibitors that we mentioned in this Review, especially KRAS^G12C^ covalent inhibitors, may be a potential treatment strategy for *KRAS*-mutant CRC.

## Emerging therapeutics

### Immune checkpoint inhibitors

Blockade of the immune checkpoint axis, such as targeting PD-L1 (programmed cell death ligand 1) or its receptor PD-1, has induced striking regressions in various malignancies. Several antibodies targeting immune checkpoint proteins have been approved by FDA and achieved a favorable clinical effect in many cancers. However, most patients with CRC, except for those with high levels of microsatellite instability (MSI) or deficient mismatch repair (dMMR), cannot therapeutically benefit from immunotherapies because they have low immunogenicity [125–127]. Furthermore, recent studies have shown that *KRAS*-mutant CRC have significantly reduced infiltration of naïve B cells, macrophage M1, activated CD4 T cells, cytotoxic cells and neutrophils, and obviously increased regulatory T cells compared to patients with *KRAS* wild-type CRC [128, 129]. Besides, multiple immune-related pathways are down-regulated in *KRAS*-mutant CRC, such as the interferon-γ (IFN-γ) pathway [129]. Therefore, these results may indicate that *KRAS*-mutant CRC harbor a more immunosuppressive microenvironment, which significantly limits the use of immune checkpoint inhibitors as monotherapy in this group of CRC patients. However, recent studies have found that treatment of KRAS^G12C^ allele-specific inhibitors or SHP2 inhibitors, or MEK inhibitors could activate anti-tumor immune cells and thereby relieve immunosuppressive status, which improves the response of *KRAS*-mutant CRC to immune checkpoint inhibitors in preclinical models [66, 87, 88, 130]. These results suggest that the combination of immune checkpoint inhibitors with these KRAS-targeted therapies is promising for the treatment of *KRAS*-mutant CRC, but the clinical efficacy of these combination therapies needs to validate in subsequent clinical trials.

### Adoptive cell therapy

Neoantigens derived from KRAS variants are regarded as “nonself” by immune system and can be recognized by antigen-specific T-cells, which make them become a potential target for immunotherapy. In a patient with metastatic *KRAS*^*G12D*^-mutant CRC, CD8+ T cells with human leukocyte antigen (HLA)-C*08:02–restricted T-cell receptors (TCRs) were found to specifically recognize mutant KRAS^G12D^. After expanding ex vivo, tumor-infiltration lymphocytes (TILs) containing approximately 75% KRAS^G12D^-specific CD8+ T cells were infused into patients. Subsequently, all seven metastatic lung lesions were observed to regress and the patient had a partial response (PR) lasting 9 months [131]. Similarly, in another study, KRAS^G12V^-mutant-specific TCRs in CD4+ T cells were identified from patients with NSCLC [132]. Moreover, a previous study immunized HLA-A*11:01 transgenic mice with mutant KRAS^G12D^ or KRAS^G12V^ peptides to generate T cells specific to these mutations. Subsequently, researchers identified the TCRs from these T cells and cloned them, and finally retrovirally transduced them into peripheral blood lymphocytes to cultivate KRAS^G12D^ or KRAS^G12V^-reactive T cells [133]. These engineering T cells were proved to inhibit KRAS^G12D^-mutant pancreatic tumor growth in a xenograft model [133]. Currently, this approach is using in two clinical trials for the treatment of patients with advanced *KRAS*^*G12D*^ or *KRAS*^*G12V*^-mutant solid tumors, including CRC (NCT03745326, NCT03190941; Table 2).

### Anti-RAS vaccines

Another emerging therapeutic is using vaccines to neutralize KRAS protein. The GI-4000 series, a kind of heat-killed recombinant *Saccharomyces cerevisiae*-derived vaccine expressing mutant RAS protein, was found to induce remission of tumors in preclinical models [134]. Furthermore, GI-4000 showed a favorable safety and immunological profile in the majority of patients with CRC in a phase I clinical trial [135]. Another approach using Targovax’s RAS peptides in combination with granulocyte-macrophage colony-stimulating factor (GM-CSF) is also able to elicit a T cell anti-tumor immune response to mutant RAS peptides [136]. TG02, Targovax’s second generation vaccine, has been studied in a phase Ib clinical trial in CRC as a monotherapy or in combination with the immune checkpoint inhibitor pembrolizumab, but the results have not been reported (NCT02933944; Tables 2 and 3). Currently, an mRNA vaccine that uses an mRNA to encode novel epitopes for common *KRAS* mutations has been developed and is able to trigger a T cell response against these mutant KRAS neo-epitopes. Meanwhile, the safety and tolerability of one such mRNA vaccine, mRNA-5671, is being evaluated in a phase I clinical trial as a monotherapy or in combination with pembrolizumab in participants with *KRAS*-mutant tumors (NSCLC, CRC, and pancreatic adenocarcinoma) (NCT03948763; Tables 2 and 3).

## Future directions and conclusions

*KRAS* is frequently mutated in CRC, and it is involved in the occurrence, progression, treatment resistance and recurrence of CRC. Considerable evidence demonstrates that *KRAS* mutations indicate a dismal prognosis for patients with CRC. The development of point mutation detection techniques has promoted the analysis of biopsy specimens and enables the analysis of plasma- and serum-containing ctDNA, allowing for early and accurate detection of *KRAS* mutations in CRC patients. Due to the presence of mutated *KRAS*, this group of CRC patients requires more precise and personalized treatment. However, historical KRAS-targeted therapies did not achieve satisfactory efficacy in patients with KRAS-mutant CRC. Recently, the development of KRAS^G12C^ allele-specific inhibitors has relighted the hope for KRAS-targeted therapies and simultaneously reveals areas for further exploration in the future.

First, more types of KRAS allele-specific inhibitors should be developed. The emergence of KRAS^G12C^ allele-specific inhibitors breaks the traditional view that KRAS is undruggable, which changes the outlook of treatment for KRAS-driven tumors. However, in CRC, KRAS^G12D^ and KRAS^G12V^ are the most common mutation subtypes and are associated with the largest patient populations (Fig. 2a). Therefore, inhibitors specific to these alleles need to be further developed. Ultimately, specific inhibitors will be required for all mutant KRAS alleles to provide more personalized treatment for patients. Notably, KRAS^G12C^ is unique among KRAS oncoproteins due to its high intrinsic GTP hydrolysis rate, which leaves more KRAS in an inactive state to facilitate KRAS^G12C^ allele-specific inhibitor binding. Therefore, solving the problem of the low GTP hydrolysis rate of other KRAS allele mutations is a challenge for satisfactory efficacy of other KRAS allele-specific inhibitors.

Second, the underlying resistance mechanisms of the drug treating *KRAS*-mutant CRC need to be elucidated. Several studies have indicated that *KRAS*-mutant CRC has an inferior response to most KRAS-targeted therapies, including KRAS^G12C^ allele-specific inhibitors, compared to NSCLC. Is the *KRAS* mutation in CRC not a driver mutation? Does the tumor have mutations in other *KRAS* alleles due to the heterogeneity of CRC? Actually, the paradigms of precision medicine in CRC have shifted from ‘one-gene, one-drug’ to ‘multi-gene, multi-drug’ and further to ‘multi-molecular, multi-drug’. Previously, an international consortium has merged six independent transcriptomic-based subtype systems that have significant interconnectivity into four consensus molecular subtypes (CMSs) with distinct characteristics: CMS1 (MSI immune); CMS2 (canonical); CMS3 (metabolic); and finally CMS4 (mesenchymal) [137]. Among these subtypes, *KRAS* mutations are enriched in CMS3 (68%), but they are also presented in other three subtypes with different proportions (23% in CMS1, 28% in CMS2, and 38% in CMS4), which indicates that *KRAS*-mutant CRC is still highly heterogeneous, and it is not advisable to rely solely on this single gene alternation to guide the treatment of CRC patients. Therefore, it is essential to integrate multi-omics data (genome, transcriptome, epigenome, metabolome and immunome) to comprehensively understand the molecular network and to refine the molecular stratification of *KRAS*-mutant CRC, which may help to develop combination therapies that are more suitable for *KRAS*-mutant CRC.

Finally, toxicity and safety should be taken into account when designing combination strategies. Historically, the efficacy of combination treatment is largely limited by toxicity, such as the combination of MEK and PI3K inhibitors. KRAS^G12C^ allele-specific inhibitors are reported to lack dose-limiting toxicities in clinical trials, which allow them to replace some of the more toxic inhibitors in combination regimens, thereby making them more tolerable to patients.

In summary, KRAS plays a critical role in the prognosis, diagnosis and treatment of CRC. The success of KRAS^G12C^ allele-specific inhibitors has pushed research on targeting KRAS to a new level, which may lead to the development of more promising KRAS-targeted approaches and provide the possibility for conquering KRAS mutations in CRC.

## Supplementary Information

**Additional file 1: Table S1.** Characteristics of KRAS targeting dugs.

## Data Availability

Not applicable.

## Abbreviations

CRC: Colorectal cancer
KRAS: Kirsten rat sarcoma
RTK: Receptor tyrosine kinase
EGFR: Epidermal growth factor receptor
GTP: Guanosine triphosphate
GDP: Guanosine diphosphate
GTPase: Guanosine triphosphatase
GEFs: Guanine exchange factors
SOS: Son of sevenless
GAPs: GTPase activating proteins
RAF: Rapidly accelerated fibrosarcoma
MEK: Mitogen-activated protein kinase kinase
ERK: Extracellular signal-regulated kinase
PI3K: Phosphatidylinositol 3-kinase
AKT: Protein kinase B
mTOR: Mechanistic target of rapamycin
OS: Overall survival
FOLFOX: 5-fluorouracil, leucovorin, and oxaliplatin
PFS: Progression-free survival
FFPE: Formalin-fixed paraffin-embedded
FNA: Fine-needle aspiration
PCR: Polymerase chain reaction
ARMS: Amplification refractory mutation system
NGS: Next generation sequencing
ctDNA: Circulating tumor DNA
cfDNA: Cell-free DNA
NSCLC: Non-small-cell lung cancer
FDA: Food and Drug Administration
PDX: Patient-derived xenografts
OR: Objective response
CDK4/6: Cyclin-dependent kinase 4/6
MAPK: Mitogen-activated protein kinase
GM-CSF: Granulocyte-macrophage colony-stimulating factor
GRB2: Growth factor receptor-bound protein 2
FTase: Farnesyltransferase
GGTase: Geranylgeranyltransferase
RCE1: RAS-converting enzyme
ICMT: Isoprenylcysteine carboxyl methyltransferase
PDEδ: Prenyl-binding protein phosphodiesterase-δ
PD-1: Programmed cell death 1
PD-L1: Programmed cell death ligand 1
MSI: Microsatellite instability
dMMR: Deficient mismatch repair
IFN-γ: Interferon-γ
TCR: T-cell receptor
TILs: Tumor-infiltration lymphocytes
PR: Partial response
CMSs: Consensus molecular subtypes
