# Supplementary material for: Role of oncogenic KRAS in the prognosis, diagnosis and treatment of colorectal cancer
Source: Mol Cancer. 2021 Nov 6;20:143. doi: 10.1186/s12943-021-01441-4 (PMC8571891; doi:10.1186/s12943-021-01441-4)
Supplement: Supplementary file 1 — Additional file 1: Table S1. Characteristics of KRAS targeting dugs. [file 12943_2021_1441_MOESM1_ESM.docx]

**Table S1.** Characteristics of KRAS targeting dugs.

| **Inhibitor** | **Target** | **Half-life** | **Administration** | **Clinical activity in *KRAS-mutant* CRC** | **Safety** | **Ref** |
| --- | --- | --- | --- | --- | --- | --- |
| AMG 510 | KRAS^G12C^ | 5.5 ±1.8 h | RP2D: 960 mg P.O daily  MTD: Not determined | Minimal to Moderate response on *KRAS^G12C^*-mutant CRC patients.  ORR: 7.1 % (3/42)  SD：66.7 % (28/42)  DCR: 73.8% (31/42) | 1. High-selectivity; well tolerance;  2. No dose-limited toxic effects were observed;  3. 56.6% (73/129) of patients had TRAEs; 11.6% (15/129) of patients have grade 3/4 events. | [12] |
| MRTX849 | KRAS^G12C^ | 24.7 h | RP2D: 600 mg P.O BID  MTD: Not determined | Minimal to moderate response on *KRAS^G12C^*-mutant CRC patients.  ORR: 16.7% (3/18)  SD: 77.7% (14/18)  DCR: 94.4% (17/18) | 1. High-selectivity; well tolerance;  2. Two grade3/4 dose-limited toxicities were observed;  3. TRAEs were primarily grade 1 events. | [68], [73] |
| BGB-283 | RAF dimmer | 15-59 h | RP2D: 30 mg P.O daily  MTD: 40mg P.O daily | No responses were observed in patients with *KRAS/NRAS*-mutant CRC (n=20). | 1. High-selectivity；  2. Dose-limited toxicities were observed;  3. TRAEs were primarily grade 1-2 events. | [105] |
| GDC-0994 | ERK | ~23 h | RP2D: 400 mg P.O daily  MTD: Not determined | SD: 20% (1/5)  PD: 80% (4/5) | 1. High-selectivity；  2. One dose-limited toxicity were observed at 600mg；  3. Grade1-4 TRAEs were observed at 400mg. | [114] |

Abbreviations: CRC, colorectal cancer; RP2D, recommended phase II dose; MTD, maximum tolerated dose; BID, twice a day; P.O, oral; ORR, objective response rate; SD, stable disease; DCR, disease control rate; PD, progressive disease; TEAEs, treatment-emergent adverse events.
